# Association between blood pressure and BMI with bladder cancer risk and mortality in 340,000 men in three Swedish cohorts

**DOI:** 10.1002/cam4.3721

**Published:** 2021-01-16

**Authors:** Stanley Teleka, Sylvia H. J. Jochems, Christel Häggström, Angela M. Wood, Bengt Järvholm, Marju Orho‐Melander, Fredrik Liedberg, Tanja Stocks

**Affiliations:** ^1^ Department of Clinical Sciences in Lund Lund University Lund Sweden; ^2^ Department of Biobank Research Umeå University Umeå Sweden; ^3^ Department of Surgical Sciences Uppsala University Uppsala Sweden; ^4^ MRC/BHF Cardiovascular Epidemiology Unit Department of Public Health and Primary Care University of Cambridge Cambridge UK; ^5^ Department of Public Health and Clinical Medicine Umeå University Umeå Sweden; ^6^ Department of Clinical Sciences in Malmö Lund University Lund Sweden; ^7^ Division of Urological Research Institution of Translational Medicine Lund University Malmö Sweden; ^8^ Department of Urology Skåne University Hospital Skåne Sweden

**Keywords:** bladder cancer, blood pressure, body mass index, confounding, survival analysis

## Abstract

**Background:**

The relation between obesity, blood pressure (BP) and bladder cancer (BC) risk and mortality remains unclear, partially due to potential confounding by smoking, the strongest risk factor for BC, and not accounting for tumor stage and grade in such studies. We investigated body mass index (BMI) and BP in relation to BC risk by stage and grade, and BC‐specific mortality, including separately among never‐smokers aimed at minimizing confounding by smoking.

**Methods:**

We analyzed 338,910 men from three Swedish cohorts, with 4895 incident BC's (940 among never‐smokers) during follow‐up. Cox regression was used to calculate hazard ratios (HR) and 95% confidence intervals adjusted for smoking status. HRs for BMI and BP were corrected for their regression dilution ratios, calculated from 280,456 individuals with 758,641 observations.

**Results:**

Body mass index was positively associated with non‐muscle invasive BC (NMIBC, HR per 5 kg/m^2^, 1.10 [1.02–1.19]) and NMIBC grade 3 (HR 1.17 [1.01–1.34]) in the full cohort, with similar effect sizes, albeit non‐significant, among never‐smokers. Systolic BP was positively associated with muscle‐invasive BC (MIBC, HR per 10 mmHg, 1.25 [1.00–1.55]) and BC‐specific mortality (HR 1.10 [1.01–1.20]) among never‐smokers, with weaker and non‐significant associations in the full cohort.

**Conclusions:**

In an analyses of BMI, BP and BC risk by stage and grade among men, we found modest positive associations between BMI and NMIBC and NMIBC grade 3. SBP was positively associated with MIBC and BC‐specific mortality in an analysis of never‐smokers, which may reflect the association, un‐confounded by smoking, also in a broader population.

## Graphical abstract

The relationships between blood pressure body (BP) mass index (BMI) and bladder cancer (BC), have shown conflicting results in previous studies, this may be due to the mixing effect of smoking in such associations and not taking into account BC characteristics. In our study of BP, BMI and BC risk among men, we attempted to remove the influence of smoking by also studying people that had never smoked and to investigate BC taking into account its characteristics such as stage and grade. We found that BMI was linked with increased risk of superficial BC, and BP with more invasive BC.

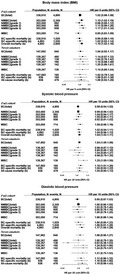

## 1 INTRODUCTION

Bladder cancer (BC) is one of the most common cancer forms in developed countries, and its relationship with metabolic risk factors including obesity, commonly measured as body mass index (BMI), and blood pressure (BP), has been inconsistent.^1^, ^2^ This may partially be due to lack of statistical power and combining sub‐groups with different etiology. With regards to BMI, a recent meta‐analysis of 14 prospective cohort studies overall showed a small, but positive non‐linear association with BC risk.^3^ However, one of the largest studies so far, (including 1391 BC cases) showed that such positive association was restricted to men,^4^ and our recent study (3737 BC cases) showed a positive association with BMI only for non‐muscle invasive BC (NMIBC).^2^ In that study, we further found positive linear associations between systolic BP (SBP) and the risk of overall BC and muscle‐invasive BC (MIBC) among men, but not women. In contrast, a recent meta‐analysis investigating associations between BP indices and overall BC risk found null associations. However, most of the included studies combined men and women in the analysis.^5^ Moreover, classification of BC aggressiveness is usually stratified based on staging in epidemiological studies. However, grading, the extent to which the tumor cells are similar in appearance and function to the normal cells, is another dimension to measure tumor aggressiveness, harboring additional information especially for NMIBC and risk of progression.^6^ Also biologically, a two‐pathway theory has been proposed with stratification in NMIBC and MIBC.^7^ Clinically, stratification in NMIBC and MIBC occurs in a majority of patients associated with preserving versus radical treatment, respectively. However, other factors such as tumor progression related to diagnostic delays confer increased risk of mortality beyond disease stratification in NMIBC and MIBC or even tumor stage.^8^

In relation to BC‐specific mortality, studies on the association with BMI and BP, respectively, have shown inconsistent results and are few.^9^, ^10^ Most of these studies were conducted among patients undergoing radical cystectomy,^11^, ^12^, ^13^, ^14^, ^15^, ^16^, ^17^, ^18^ and fewer studies were conducted at population level.^19^, ^20^ Most studies investigated associations either from the time of study enrollment (in the full population), or from the time of diagnosis (among cases only), but not both. Investigating either time‐line has advantages, but may also introduce certain biases that could be mitigated by investigating both time‐lines.^21^, ^22^, ^23^, ^24^

Smoking is the strongest known risk factor for BC and accounts for up to 50% of the cases.^25^ Sufficiently accounting for such a strong risk factor in observational studies is difficult, and residual confounding may persist. Investigating risk factors in relation to BC risk separately among never‐smokers may disentangle the risk factor association from smoking; however, this may be difficult to accomplish due to the challenge of achieving sufficient statistical power in an analysis restricted to never‐smokers.

The aim of the study was to investigate the associations between BMI and BP and BC risk, for BC overall and separately for NMIBC and MIBC and by tumor grade. Furthermore, we aimed to investigate the association between BMI and BP and BC‐specific mortality from the time of study enrollment and, among cases, from the time of diagnosis with additional adjustment for clinical characteristics. We investigated all associations in the full cohort and separately among never‐smokers. Due to the population composition, we focused our analysis on men.

## 2 MATERIALS AND METHODS

### 2.1 Study population

The study included participants from three prospective Swedish cohorts, the Västerbotten Intervention Programme (VIP), the Malmö Preventive Project (MPP) and the Construction Workers Cohort (CWC), of which a detailed description has been published elsewhere.^26^, ^27^, ^28^

### 2.2 Exposure assessment

Height and weight were measured with individuals wearing no shoes and light clothing.^29^, ^30^ BP was taken in a supine position using a standard mercury sphygmomanometer in all the cohorts; in the VIP and CWC a single reading was taken after 5 min of rest, and in the MPP, BP was recorded after an average of 2 readings taken with a 10 min interval.

### 2.3 Follow‐up and end point assessment

Any cancer diagnosis, death and its cause, and migration status were identified through linkage of each individual's unique identification number with Sweden's National Cancer Register, Cause of Death Register and Population Register, respectively, for events up until 31 December 2014. BC was defined according to the 10th version of the International Classification of Diseases (ICD‐10) code C67 (0–9), including carcinoma in situ (D09.0). The Swedish National Register for Bladder Cancer register was used for the classifications of BC into NMIBC and MIBC, and this started in 1997, a detailed description on how BC tumors were classified by stage and grade is described in the Supporting Information (Text S1). Death due to BC (BC‐specific mortality) was defined as BC (ICD‐10, C67) reported as the underlying cause of death in the Swedish national cause of death registry.

### 2.4 Selection criteria

The study population was initially composed of 521,896 individuals with 1,342,110 observations from the health examination data, out of which 338,910 individuals, each individual with one baseline observation, were included for the final analysis (Figure S1). For individuals with repeated observations, the first was selected as the baseline observation. Out of the 182,986 excluded individuals, the most common causes of exclusion were those younger than 20 years, women, missing smoking data, and those with any prevalent cancer which was defined as any malignant neoplasm, including malignant neoplasms of hematopoietic or lymphoid origin and other related tissues (ICD‐10, C81‐C96), but excluding basaliomas and all carcinomas in situ.

### 2.5 Statistical analysis

Cox proportional hazards regression was used to calculate hazard ratios (HRs) with their 95% confidence intervals (95% CI) to investigate the risk of BC end‐points by levels of BMI, SBP, and diastolic BP (DBP) in the full cohort, and additionally among never‐smokers, using age as the underlying time scale. Participants were followed from the date of baseline examination, up until the date of event of interest or until censoring due to the diagnosis of another cancer, emigration and death, or until the end of follow‐up, whichever one came first. Follow‐up of NMIBC and MIBC began on 1 January 1997, and 35,860 individuals who were censored before that date were excluded from the analysis. We adjusted the analyses for smoking status (three categories), age at baseline examination (continuous), date of birth (five categories), cohort (three categories), level of education (eight categories), and BMI ([quartiles] except for the analysis of BMI). Additionally, the metabolic factors were investigated in categories of BMI, SBP and DBP in relation with BC end‐points. Test for trends in these categories were performed by regressing the BC outcomes against the average in each category. We additionally investigated the shape of association between BMI (per 5 kg/m^2^), SBP and DBP (per 10 mmHg), and BC end‐points using restricted cubic spline regression, and we tested for the linearity of these associations with the likelihood ratio test (Figure S2–S6).

In the case‐only analysis, we investigated the associations between BMI, BP, and BC‐specific mortality and all‐cause mortality using Cox regression with follow‐up time from the date of BC diagnosis as the underlying time scale. The same adjustments were used as in the full cohort analysis; however, instead of adjusting for age at baseline examination, we adjusted for age at diagnosis and additionally for tumor grade (four categories) and stage (three categories), type of treatment (BC‐specific [eight categories]) and for the Charlson co‐morbidity index (four categories).^31^ A detailed description of the categorical variables used in models is found in in the supplements (Table S1).

We calculated Schoenfield residuals for exposures and co‐variables to test for the proportional hazards assumption in the Cox models. Depending on the end‐point being analyzed, one or two co‐variables were suggestive of violating this assumption; however, including them as strata in the Cox models did not materially change the HRs, thus, they were retained within the models as co‐variables rather than in strata.

We corrected the HRs for intra‐personal variability and measurement error using a RDR based method as described by Wood et al.^32^ The values for the calculated RDRs are shown in Table S2. All HRs were corrected for RDR by: HR_corrected_ = exponent^(log [HR original]/RDR)^. The corrected HRs are interpreted as the expected HRs of “usual” adult level of BMI and BP, respectively.

We performed all the statistical analyses in STATA 13, (StataCorp LLC).

## 3 RESULTS

Table 1 shows the characteristics of the study participants according to smoking status. There were 147,692 never‐smokers, 64,492 ex‐smokers and 126,726 current smokers. On average, never‐smokers, had a BMI of 24.8 (SD = 3.3), while ex‐smokers had a BMI 25.3 (SD = 3.3) and current smokers had a BMI of 24.3 (SD = 3.2). With regards to BP, never‐smokers had an average SBP of 131 mmHg (SD = 15) and an average DBP of 80 mmHg (SD = 11) and the corresponding values among ex‐smokers and current smokers were 134 mmHg (SD = 16)/83 mmHg (SD = 10) and 133 mmHg (SD = 15)/81 mmHg (SD = 10), respectively. A breakdown of study participant characteristics according to cohort is shown in Table S3. During an average follow‐up of 28 years, 4895 men had been diagnosed with BC, of which 1020 had died from BC. The associations between BMI, BP, and BC outcomes were approximately linear except for the association between BMI and overall BC, and SBP and MIBC (Figure S2–S6). There was a positive association between BMI levels above 25 kg/m^2^ and BC (HR per 5 kg/m^2^, 1.15 [95% CI, 1.03–1.26]), and a suggestive, non‐significant, association between SBP in the lower range and risk of MIBC.

**TABLE 1:** Characteristics of the 338,910 men in the study according to smoking status

Characteristic	Never‐smokers (*n* = 147,692)	Ex‐smokers (*n* = 64,492)	Current Smokers (*n* = 126,726)	Total (*n* = 338,910)
Cohort, *n* (%)				
Västerbotten Intervention Programme	31,922 (21.6)	11,331 (17.6)	8802 (7.0)	52,055 (15.3)
Malmö Preventive Project	7588 (5.1)	3568 (5.5)	11,120 (8.8)	22,276 (6.6)
Construction Workers Cohort	108,182 (73.3)	49,593 (76.9)	106,804 (84.2)	264,579 (78.1)
Baseline age, years, mean (SD)	36.3 (12.4)	42.2 (12.3)	38.9 (12.3)	38.4 (12.5)
BMI, kg/m^2^, mean (SD)	24.8 (3.3)	25.3 (3.3)	24.3 (3.2)	24.7 (3.3)
Category of BMI, kg/m^2^, *n* (%)				
<18.5	1023 (0.7)	341 (0.5)	1667 (1.3)	3031 (0.9)
18.5–24.9	85,385 (57.8)	32,360 (50.2)	78,316 (61.8)	196,061 (57.9)
25–29.9	51,482 (34.9)	26,438 (41.0)	40,031 (31.6)	117,951 (34.8)
≥30	9802 (6.6)	5353 (8.3)	6712 (5.3)	21,867 (6.4)
Systolic BP, mmHg, mean (SD)	131.1 (14.8)	134.1 (15.8)	132.5 (15.0)	132.2 (15.1)
Diastolic BP, mmHg, mean (SD)	79.7 (10.5)	82.5 (10.3)	81.0 (10.2)	80.7 (10.4)
Category of systolic/diastolic BP, *n* (%)				
<140/90 mmHg	97,836 (66.2)	36,783 (57.0)	78,256 (61.8)	212,875 (62.8)
140/90–159/99 mmHg	39,845 (27.0)	21,093 (32.7)	38,672 (30.5)	99,610 (29.4)
≥160/100 mmHg	10,011 (6.8)	6616 (10.3)	9798 (7.7)	26,425 (7.8)
Follow‐up, years, mean (SD)^a^	27.1 (10.8)	28.9 (11.1)	28.8 (10.5)	28.1 (10.8)
Incident cases of BC overall, *n*	940	1148	2807	4895
Level of invasion, *n* (%)^b^				
Non‐muscle invasive	490 (78.3)	560 (76.3)	1305 (76.4)	2355 (76.7)
Muscle invasive	136 (21.7)	174 (23.7)	404 (23.6)	714 (23.3)
Grade among NMIBC, *n* (%)^c^				
Grade 1	138 (29.7)	134 (25.5)	410 (32.7)	682 (30.4)
Grade 2	185 (39.8)	216 (41.1)	504 (40.2)	905 (40.3)
Grade 3	142 (30.5)	176 (33.4)	340 (27.1)	658 (29.3)

Abbreviations: BC, bladder cancer; BMI, body mass index; BP, blood pressure; NMIBC, non‐muscle invasive BC; SD, standard deviation.

^a^ Follow‐up until bladder cancer risk or censoring, last date of follow‐up was 31 December 2014.

^b^ Out of the 4895 incident bladder cancer cases, staging data were available for 3069 cases, the remaining 1826 cases either occurred before 1997 before staging data were available or staging data were available but stage could not be determined.

^c^ Out of the 2355 incident NMIBC cases grading data were available for 2245 cases, for the remaining 110 cases, grade could not be determined.

**FIGURE 1:**
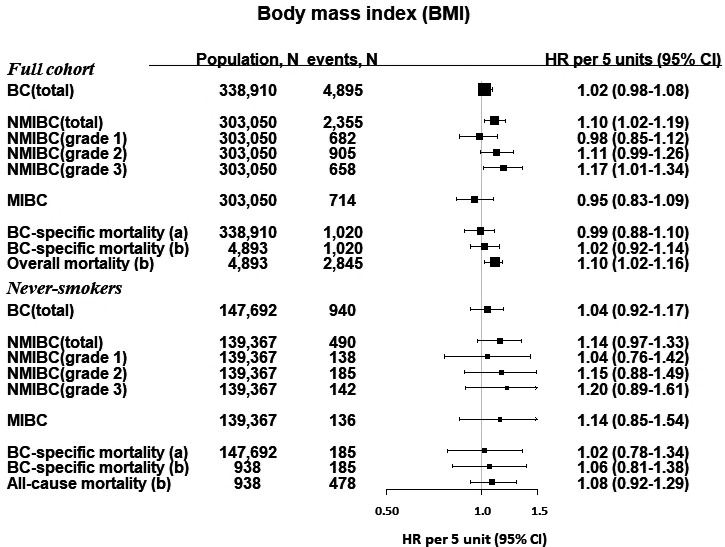
Hazard ratios and 95% confidence intervals for BC outcomes per 5 kg/m^2^ increment of BMI. For BC‐specific mortality, we investigated associations for (A) the time of study enrollment, and (B) among cases, from the time of diagnosis. BC, bladder cancer; kg, kilogram; m, meter; BMI, body mass index; NMIBC, non‐muscle invasive bladder cancer; MIBC, muscle‐invasive bladder cancer. ^†^Data on tumor characteristics were only available from 1997, therefore, the analysis for NMIBC (and by grade) and MIBC only began from 1 January 1997 and any diagnosis of BC or other censoring events (diagnosis of other cancers, emigration or death) were excluded from this analysis (35,860 participants). Two incident bladder cancer cases were excluded in the mortality analysis

**FIGURE 2:**
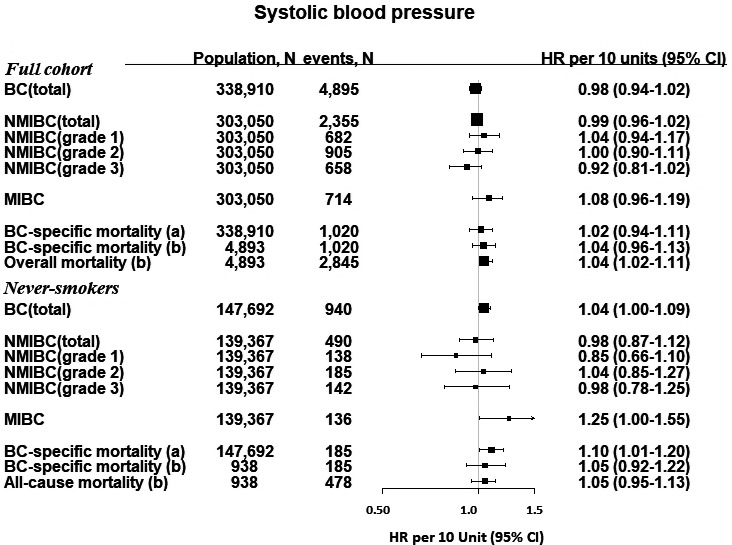
Hazard ratios and 95% confidence intervals for BC outcomes per 10 mmHg increment of systolic blood pressure. For BC‐specific mortality, we investigated associations for (A) the time of study enrollment, and (B) among cases, from the time of diagnosis. BC, bladder cancer; mmHg, millimeters of mercury; NMIBC, non‐muscle invasive bladder cancer; MIBC, muscle‐invasive bladder cancer. ^†^Data on tumor characteristics were only available from 1997, therefore, the analysis for NMIBC (and by grade) and MIBC only began from 1 January 1997 and any diagnosis of BC or other censoring events (diagnosis of other cancers, emigration or death) were excluded from this analysis (35,860 participants). Two incident bladder cancer cases were excluded in the mortality analysis

**FIGURE 3:**
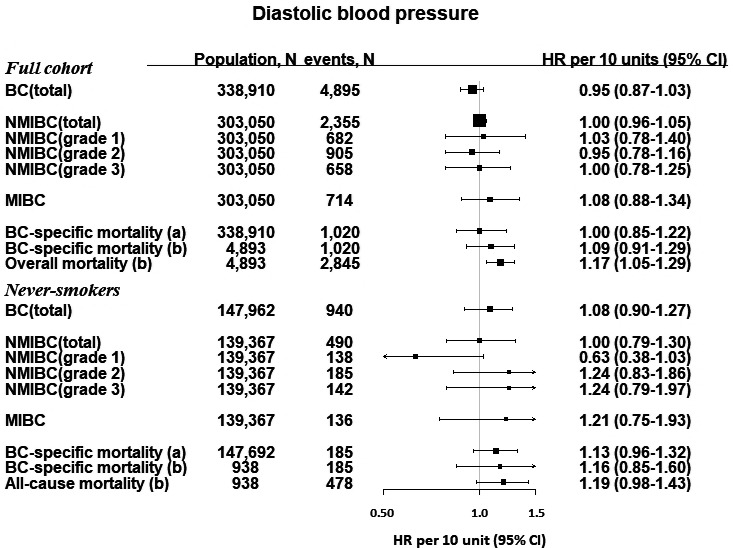
Hazard ratios and 95% confidence intervals for BC outcomes per 10 mmHg increment of diastolic blood pressure. For BC‐specific mortality, we investigated associations for (A) the time of study enrollment, and (B) among cases, from the time of diagnosis. BC, bladder cancer; mmHg, millimeters of mercury; NMIBC, non‐muscle invasive bladder cancer; MIBC, muscle‐invasive bladder cancer. ^†^Data on tumor characteristics were only available from 1997, therefore, the analysis for NMIBC (and by grade) and MIBC only began from 1 January 1997 and any diagnosis of BC or other censoring events (diagnosis of other cancers, emigration or death) were excluded from this analysis (35,860 participants). Two incident bladder cancer cases were excluded in the mortality analysis

Hazard ratios and 95% CIs of BC outcomes per continuous increase in BMI and BP are shown in Figures 1, 2, 3. BMI was positively associated with risk of all NMIBC (HR per 5 kg/m^2^, 1.10 [95% CI, 1.02–1.19]) and with NMIBC grade 3 risk (HR per 5 kg/m^2^, 1.17 [95% CI, 1.01–1.34]). The effect sizes in these associations were similar, but did not reach significance in an analysis including only never‐smokers. There were no other statistically significant associations between BMI and BC outcomes. BP was not associated with BC outcomes in the full cohort; however, among never‐smokers, SBP was positively associated with MIBC (HR per 10 mmHg, 1.25 [95% CI, 1.00–1.55], and with BC‐specific mortality in the baseline‐to‐event analysis (HR per 10 mmHg, 1.10 [1.01–1.20]), but not in the case‐only analysis (HR per 10 mmHg, 1.05 [0.92–1.22]).

There were positive associations between BMI, SBP, and DBP, and risk of all‐cause mortality, which did not reach significance in the analyses of never‐smokers only (Figures 1, 2, 3). Categories of BMI and BP in relation with BC risk and mortality largely reflected the associations reported as forest plots and restricted cubic splines (Table S4–S5).

## 4 DISCUSSION

In this large prospective study of nearly 340,000 men including 4900 incident BC cases, we found positive associations between BMI and NMIBC risk, especially high‐grade tumors, and between SBP and MIBC among never‐smokers, which are expected to display little or no confounding by smoking and may depict the smoking un‐confounded association also in a broader population. SBP was further positively associated with BC‐specific mortality among never‐smokers, otherwise, no clear associations were observed for BMI, BP, and BC.

The association between BMI and NMIBC observed in this study was similar to the finding in our recent study,^2^ which has some overlap with the present study, with the VIP and MPP comprising around 20% of the respective study populations. We investigated NMIBC and MIBC as two distinct entities; however, this separation may be too simplified. The relationship between NMIBC and MIBC may also be seen as a continuum, and while a majority of NMIBC are made up of low grade Ta tumors that rarely progress to severe forms, high grade NMIBC are more likely to progress into MIBC. The classification of such tumors into NMIBC and MIBC thus is affected by the time of capture, that is, diagnostic delay. In the present study, we were able to divide NMIBCs by tumor grade and found a positive linear association between BMI and NMIBC grade 3 risk. Roswall et al., in a similar analysis albeit, with fewer cases, found no such association.^4^ It remains unclear why we observe an association with NMIBC and not with MIBC, and biological mechanisms linking BMI and BC also remain unclear.

We found no association between BMI and BC‐specific mortality. This result remains a source of controversy^9^, ^10^ as some studies found a positive association,^11^, ^12^, ^13^, ^14^ while others found a null association.^15^, ^16^, ^17^, ^18^, ^19^, ^20^ One reason for inconsistent results may partially be differences in study design. Whereas most studies were conducted retrospectively and within a clinical setting,^11^, ^12^, ^13^, ^14^ a few studies were conducted prospectively, and these found no association.^19^, ^20^ Additionally, some studies analyzed men and women together, despite higher BC incidence rates among men (3:1 ratio)^1^ and a poorer prognosis among women,^33^ and their potentially differential BC etiology. Further large, well‐designed and sufficiently powered studies are needed to investigate BC‐specific mortality in relevant sub‐groups.

We found an overall positive association between SBP and MIBC among never‐smokers, which was weaker and non‐significant in the full cohort. Again, these findings are consistent with our previous study, where we additionally found an association with DBP.^2^ The association between BP (especially SBP) and MIBC and not with NMIBC, suggests that BP might play a role in BC progression as opposed to BC initiation. In further support of such hypothesis, we found a positive association between SBP and BC‐specific mortality among never‐smokers in this study, and positive associations with both SBP and DBP in our previous study. The association between SBP and MIBC could be influenced by participants being managed for hypertension in the health care system likely undergoing further tests that may lead to early detection of BC, however, such detection bias, if substantial, would also lead to an association between SBP and NMIBC, which was not found in this study.

The large study size, the virtually complete follow‐up in the Swedish registers, and the RDR correction were the main strengths of our study. The large sample size enabled us to conduct analyses in different sub‐groups, including analysis by tumor grade and among never‐smokers only. An exposure measured on a single occasion is prone to random error due to technical error, and short‐term and long‐term intra‐individual variability, which results in dilution of the exposure‐outcome association, that is, regression dilution bias. By correcting for this bias, we investigated the association between the “usual” levels of the exposure and the outcome, which was particularly important in this study due to long follow‐up. We also investigated BC‐specific mortality using two approaches. In the first approach we used the full‐cohort and follow‐up was from the date of baseline examination. Results from this analysis reflect the influence of the metabolic factors on both the incidence and survival of BC, however, they are unlikely to suffer from selection bias.^23^, ^24^ In the second approach, we analyzed the survival of BC cases, which allowed us to adjust for tumor characteristics, co‐morbidities and types of BC‐related treatment; factors that have a large bearing on BC‐specific survival. However, this last approach is prone to a type of selection bias called collider bias,^23^, ^24^ which may occur if the exposure is related to the risk of BC, which could explain the weaker and non‐significant finding for SBP and BC‐specific mortality in the case‐only analysis as compared to in the baseline‐to‐event analysis.

The study had several limitations. First, there was no data on anti‐hypertensive medication which have overall shown a positive association with BC^34^ and might modify or mediate the association between BP and BC. Our results for BC‐specific mortality should be interpreted with caution, as these were rather inconsistent and based on small numbers, especially among never‐smokers. Lastly, the many tests performed in the analysis, may potentially attribute the significant findings to chance alone.

In conclusion, we found a positive association between BMI and NMIBC and particularly between BMI and NMIBC grade 3, and between SBP and MIBC among never‐smokers. Additionally, we found a positive association between SBP and BC‐specific mortality among never‐smokers. The findings on grade and among never‐smokers underscore the importance of additionally investigating grade in the assessment of tumor aggressiveness, and the importance of minimizing the influence of smoking, such as analyzing never‐smokers only, ideally in even larger populations than ours.

## CONFLICTS OF INTEREST

None declared.

## AUTHOR CONTRIBUTIONS

Stanley Teleka: Conceptualization, Data curation, Formal analysis, Writing‐original draft and Writing‐review and editing. Tanja Stocks: Funding acquisition, Conceptualization, Writing‐original draft and Writing‐review and editing. Sylvia H J Jochems: Data curation and Writing‐review and editing. Angela M Wood: Writing‐review and editing. Bengt Järvholm: Writing‐review and editing. Marju Orho‐Melander: Writing‐review and editing. Fredrik Liedberg: Writing‐review and editing.

## ETHICS APPROVAL

The ethics committee at Umeå University approved the study (no. 2012‐354‐31M, 2014‐162‐32M, 2014‐267‐32M and 2015‐7‐32M). This study was performed in accordance with the Declaration of Helsinki.

## Supporting information

Supplementary Material

## Data Availability

The data that support the findings of this study are available from https://www.malmo‐kohorter.lu.se/ for the Malmö Preventive Project, from https://www.umu.se/en/biobank‐research‐unit/research/northern‐sweden‐health‐and‐disease‐study‐vip‐monica‐and‐the‐mammography‐screening‐project/ for the VIP, and from https://www.umu.se/en/research/projects/work‐retirement‐and‐health/ for the CwC, with ethical approval and permission from the respective steering committee through registration and study approval. Restrictions apply to the availability of the data, appropriate procedures were followed to obtain data for this study.
