# Supplementary material for: Association between blood pressure and BMI with bladder cancer risk and mortality in 340,000 men in three Swedish cohorts
Source: Cancer Med. 2021 Jan 16;10(4):1431–8. doi: 10.1002/cam4.3721 (PMC7926028; doi:10.1002/cam4.3721)
Supplement: Supplementary file 1 — Supplementary Material [file CAM4-10-1431-s001.pdf]

SUPPLEMENTARY MATERIAL

Summary

Supplementary text S1

Figure S1

Figure S2

Figure S3

Figure S4

Figure S5

Figure S6

Table S1

Table S2

Table S3

Table S4

Table S5

Supplementary text S1

Depth of tumor invasion was based on the T-stage from the 1987, 1997, 2002 and 2009 versions of TNM classification. The T-stage was clinically determined based on histology from tumor biopsies, palpation and radiological findings. For this study, tumors were further classified into two groups based on their T-stage, NMIBC included non-invasive tumors (Ta), carcinoma in situ (Tis) and tumors that have not invaded beyond the lamina propria (T1). MIBC included tumors that have invaded the muscularis propria (T2) to tumors that have invaded the pelvic and/or abdominal wall (T4b), and metastatic tumors (distal [M1] and/or lymph node spread [from N1]) were also included in this group. Tumors were also stratified into 3 groups based on the 1973 (from 1997-2002) and 1999 (from 2003 onwards) WHO grading. The 1973 grading system included G1, G2 and G3, the 1999 grading system was similar, but additionally included papillary urothelial neoplasm of low malignant potential (PUNLMP), a tier before G1[29], due to a small number of cases for PUNLMP, they were combined with G1 in the analysis. Tumor data were available for 93% of all cases diagnosed from 1 January 1997, the inception date for the Swedish National Register for Urinary Bladder Cancer. Out of the 4,895 incident BC cases, 246 (5%) occurred within 5 years after baseline examination. Death due to BC was defined as BC (ICD-10, C67) reported as the underlying cause of death in the Swedish national cause of death registry.

Figure S1: Selection of the participants

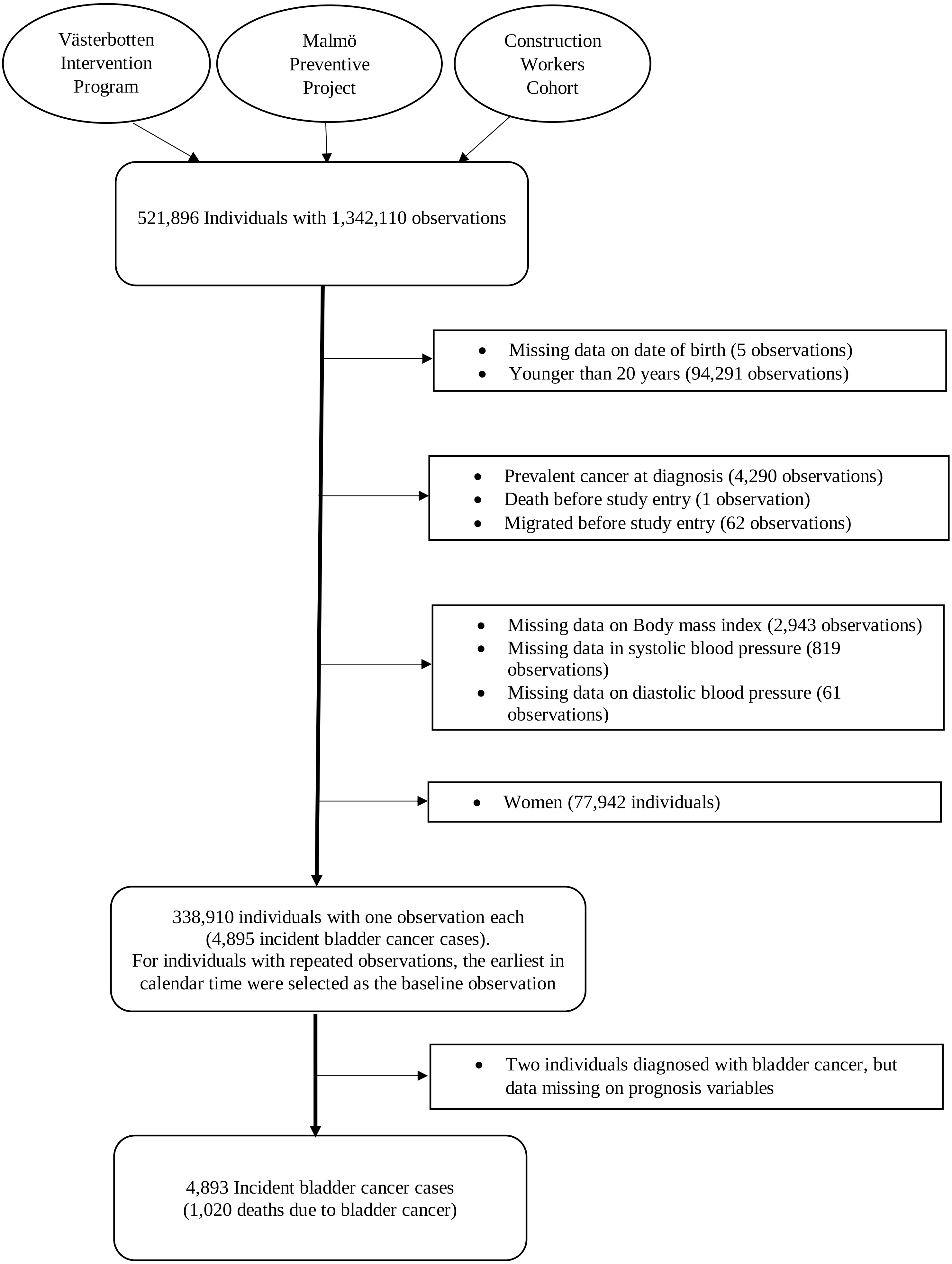

Figure S2: The Hazard ratio (HR) (black line) and 95% confidence interval (shaded area) of BC outcomes by per 5Kg/m^2 of BMI among men in the full population (A) and among never-smokers only (B). Models were derived from restricted cubic spline regression, with knots placed at percentiles of 5, 35, 65, and 95. Participants who had values that were more extreme than +/-2.5 standard deviations (SD) of calculated Z-scores were excluded from the analyses. P value LR test, linear-spline, refers to likelihood-ratio (LR) tests of the linear model nested in a model with the addition of splines. BMI indicates body mass index.

A)

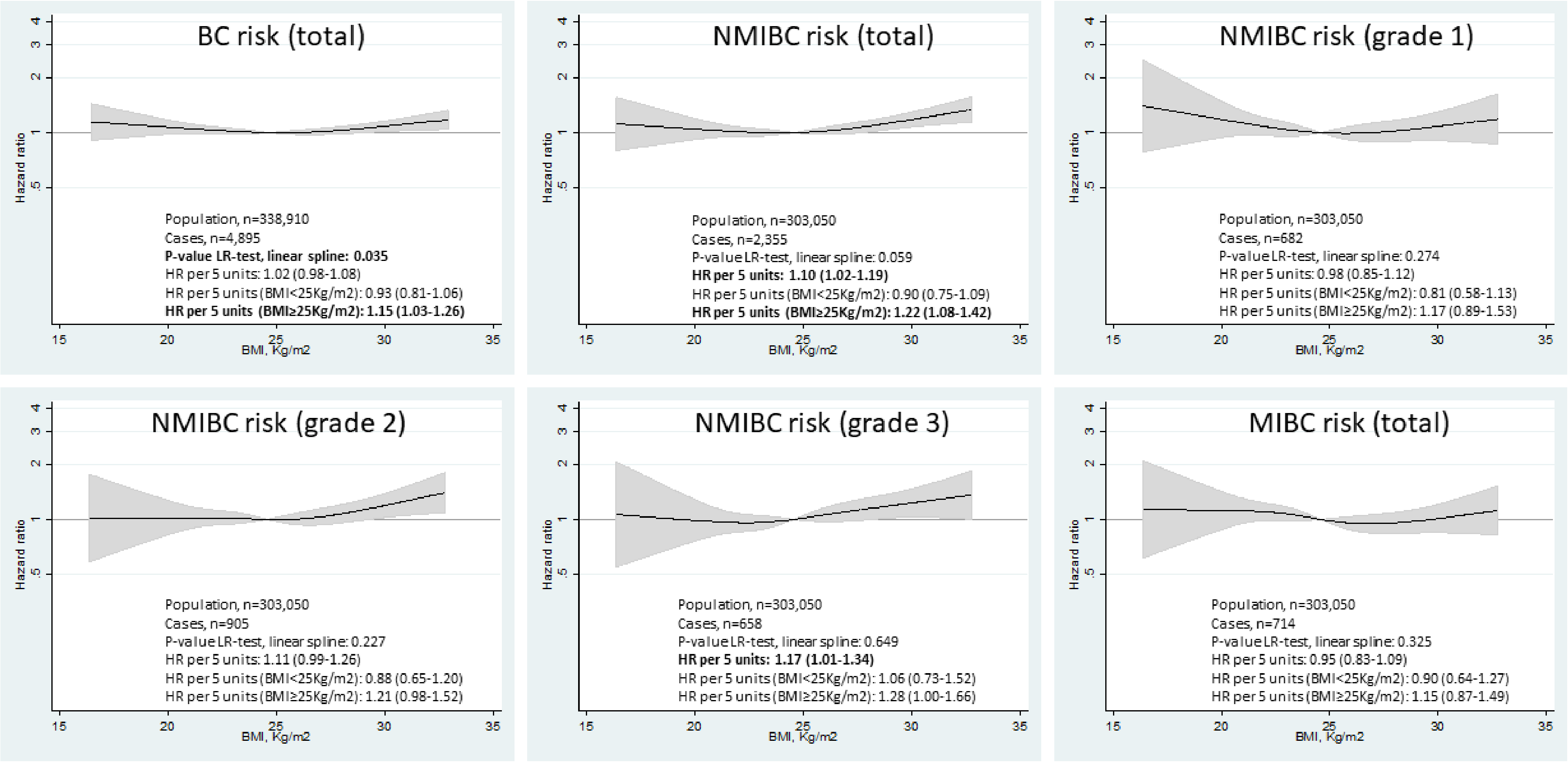

B)

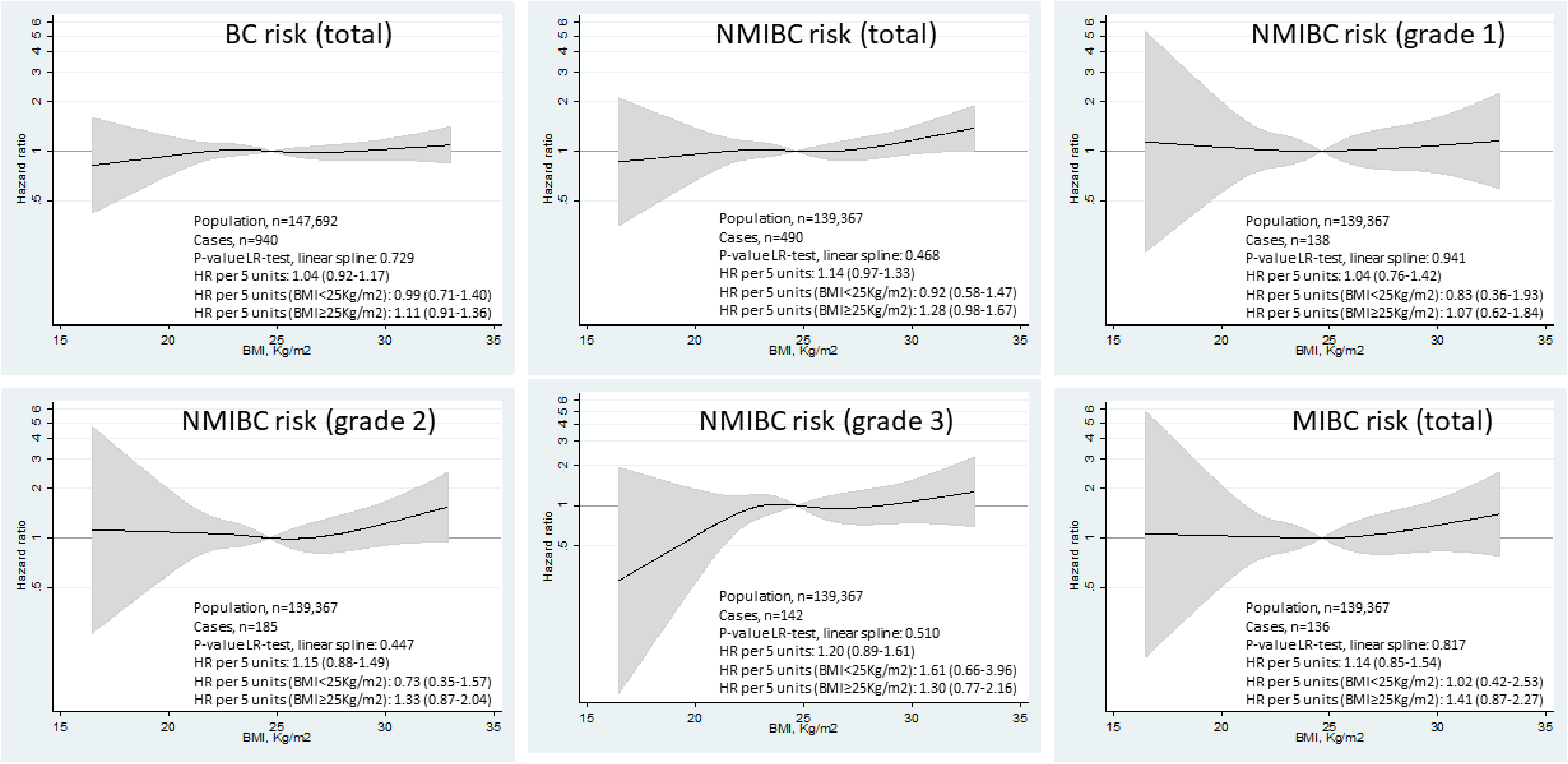
BC risk (total)
Population, n=147,692
Cases, n=940
P-value LR-test, linear spline: 0.729
HR per 5 units: 1.04 (0.92-1.17)
HR per 5 units (BMI<25Kg/m2): 0.99 (0.71-1.40)
HR per 5 units (BMI≥25Kg/m2): 1.11 (0.91-1.36)
NMIBC risk (total)
Population, n=139,367
Cases, n=490
P-value LR-test, linear spline: 0.468
HR per 5 units: 1.14 (0.97-1.33)
HR per 5 units (BMI<25Kg/m2): 0.92 (0.58-1.47)
HR per 5 units (BMI≥25Kg/m2): 1.28 (0.98-1.67)
NMIBC risk (grade 1)
Population, n=139,367
Cases, n=138
P-value LR-test, linear spline: 0.941
HR per 5 units: 1.04 (0.76-1.42)
HR per 5 units (BMI<25Kg/m2): 0.83 (0.36-1.93)
HR per 5 units (BMI≥25Kg/m2): 1.07 (0.62-1.84)
NMIBC risk (grade 2)
Population, n=139,367
Cases, n=185
P-value LR-test, linear spline: 0.447
HR per 5 units: 1.15 (0.88-1.49)
HR per 5 units (BMI<25Kg/m2): 0.73 (0.35-1.57)
HR per 5 units (BMI≥25Kg/m2): 1.33 (0.87-2.04)
NMIBC risk (grade 3)
Population, n=139,367
Cases, n=142
P-value LR-test, linear spline: 0.510
HR per 5 units: 1.20 (0.89-1.61)
HR per 5 units (BMI<25Kg/m2): 1.61 (0.66-3.96)
HR per 5 units (BMI≥25Kg/m2): 1.30 (0.77-2.16)
MIBC risk (total)
Population, n=139,367
Cases, n=136
P-value LR-test, linear spline: 0.817
HR per 5 units: 1.14 (0.85-1.54)
HR per 5 units (BMI<25Kg/m2): 1.02 (0.42-2.53)
HR per 5 units (BMI≥25Kg/m2): 1.41 (0.87-2.27)
Hazard ratio
BMI, Kg/m2
15
20
25
30
35
.5
1
2
3
4
5
6

Figure S3: The Hazard ratio (HR) (black line) and 95% confidence interval (shaded area) of BC outcomes by per 10 mmHg of systolic blood pressure among men in the full population (A) and among never-smokers only (B). Models were derived from restricted cubic spline regression, with knots placed at percentiles of 5, 35, 65, and 95. Participants who had values that were more extreme than the equivalent of +/-2.5 standard deviations (SD) were excluded from the analyses. P value LR test, linear-spline, refers to likelihood-ratio (LR) tests of the linear model nested in a model with the addition of splines.

A)

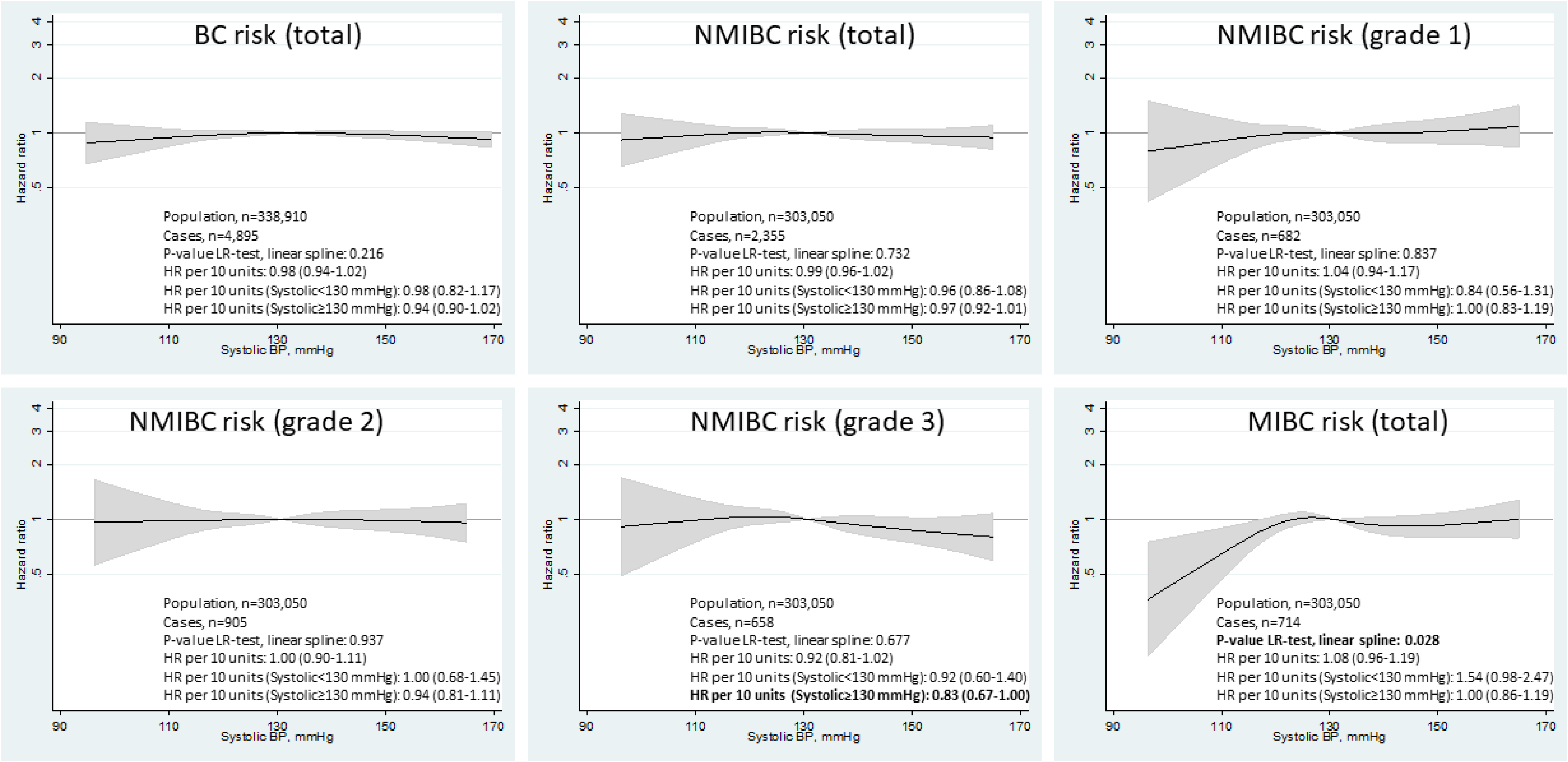

B)

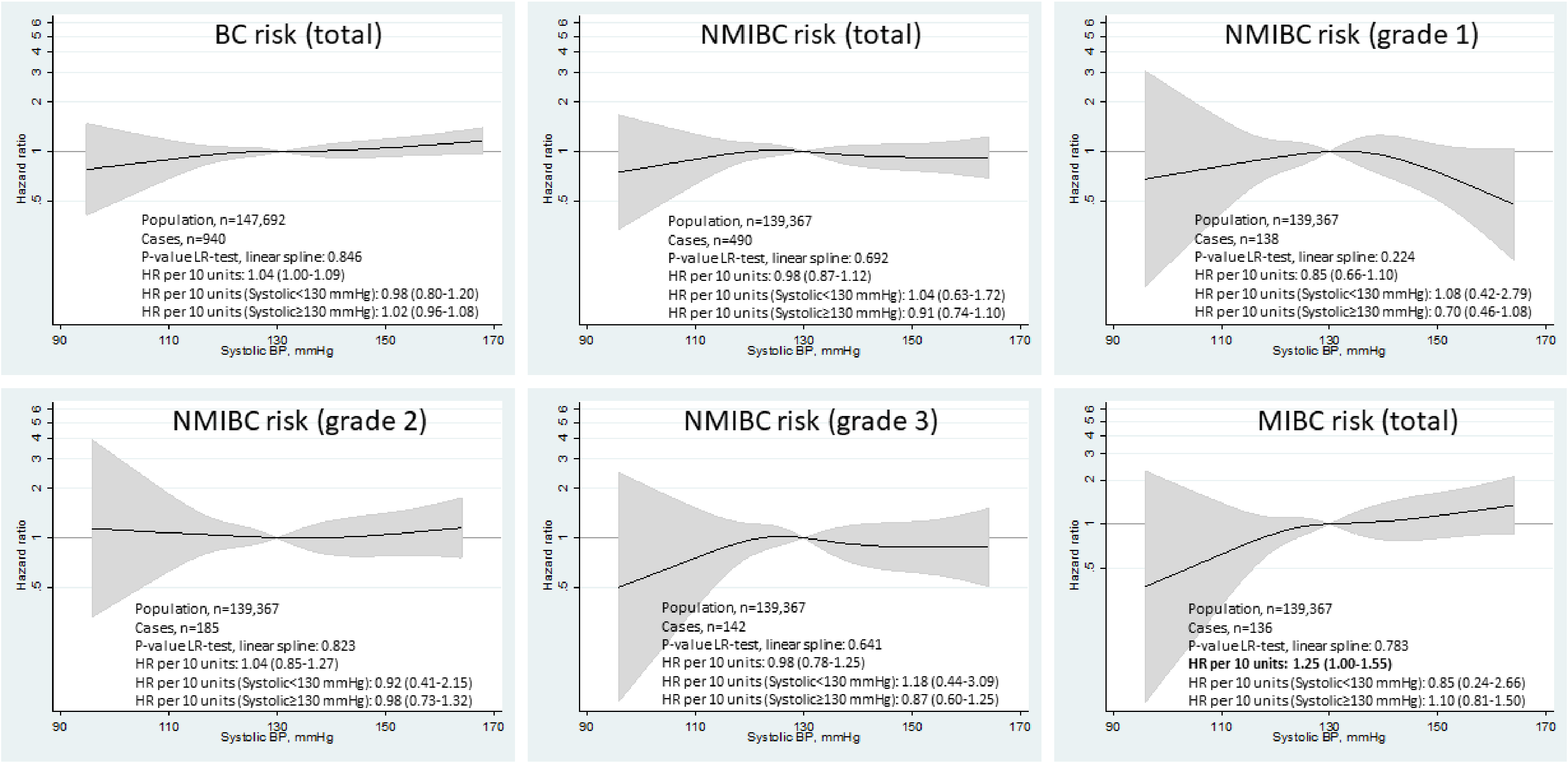
BC risk (total)
Hazard ratio
Population, n=147,692
Cases, n=940
P-value LR-test, linear spline: 0.846
HR per 10 units: 1.04 (1.00-1.09)
HR per 10 units (Systolic<130 mmHg): 0.98 (0.80-1.20)
HR per 10 units (Systolic≥130 mmHg): 1.02 (0.96-1.08)
Systolic BP, mmHg
NMIBC risk (total)
Population, n=139,367
Cases, n=490
P-value LR-test, linear spline: 0.692
HR per 10 units: 0.98 (0.87-1.12)
HR per 10 units (Systolic<130 mmHg): 1.04 (0.63-1.72)
HR per 10 units (Systolic≥130 mmHg): 0.91 (0.74-1.10)
NMIBC risk (grade 1)
Population, n=139,367
Cases, n=138
P-value LR-test, linear spline: 0.224
HR per 10 units: 0.85 (0.66-1.10)
HR per 10 units (Systolic<130 mmHg): 1.08 (0.42-2.79)
HR per 10 units (Systolic≥130 mmHg): 0.70 (0.46-1.08)
NMIBC risk (grade 2)
Population, n=139,367
Cases, n=185
P-value LR-test, linear spline: 0.823
HR per 10 units: 1.04 (0.85-1.27)
HR per 10 units (Systolic<130 mmHg): 0.92 (0.41-2.15)
HR per 10 units (Systolic≥130 mmHg): 0.98 (0.73-1.32)
NMIBC risk (grade 3)
Population, n=139,367
Cases, n=142
P-value LR-test, linear spline: 0.641
HR per 10 units: 0.98 (0.78-1.25)
HR per 10 units (Systolic<130 mmHg): 1.18 (0.44-3.09)
HR per 10 units (Systolic≥130 mmHg): 0.87 (0.60-1.25)
MIBC risk (total)
Population, n=139,367
Cases, n=136
P-value LR-test, linear spline: 0.783
HR per 10 units: 1.25 (1.00-1.55)
HR per 10 units (Systolic<130 mmHg): 0.85 (0.24-2.66)
HR per 10 units (Systolic≥130 mmHg): 1.10 (0.81-1.50)

Figure S4: The Hazard ratio (HR) (black line) and 95% confidence interval (shaded area) of BC-specific mortality and all-cause mortality by per 5Kg/m^2 of BMI (A) and per 10 mmHg of systolic blood pressure (B) among men in the full population and among never-smokers only. Models were derived from restricted cubic spline regression, with knots placed at percentiles of 5, 35, 65, and 95. Participants who had values that were more extreme than the equivalent of +/-2.5 standard deviations (SD) were excluded from the analyses. P value LR test, linear-spline, refers to likelihood-ratio (LR) tests of the linear model nested in a model with the addition of splines. BMI indicates body mass index

A)

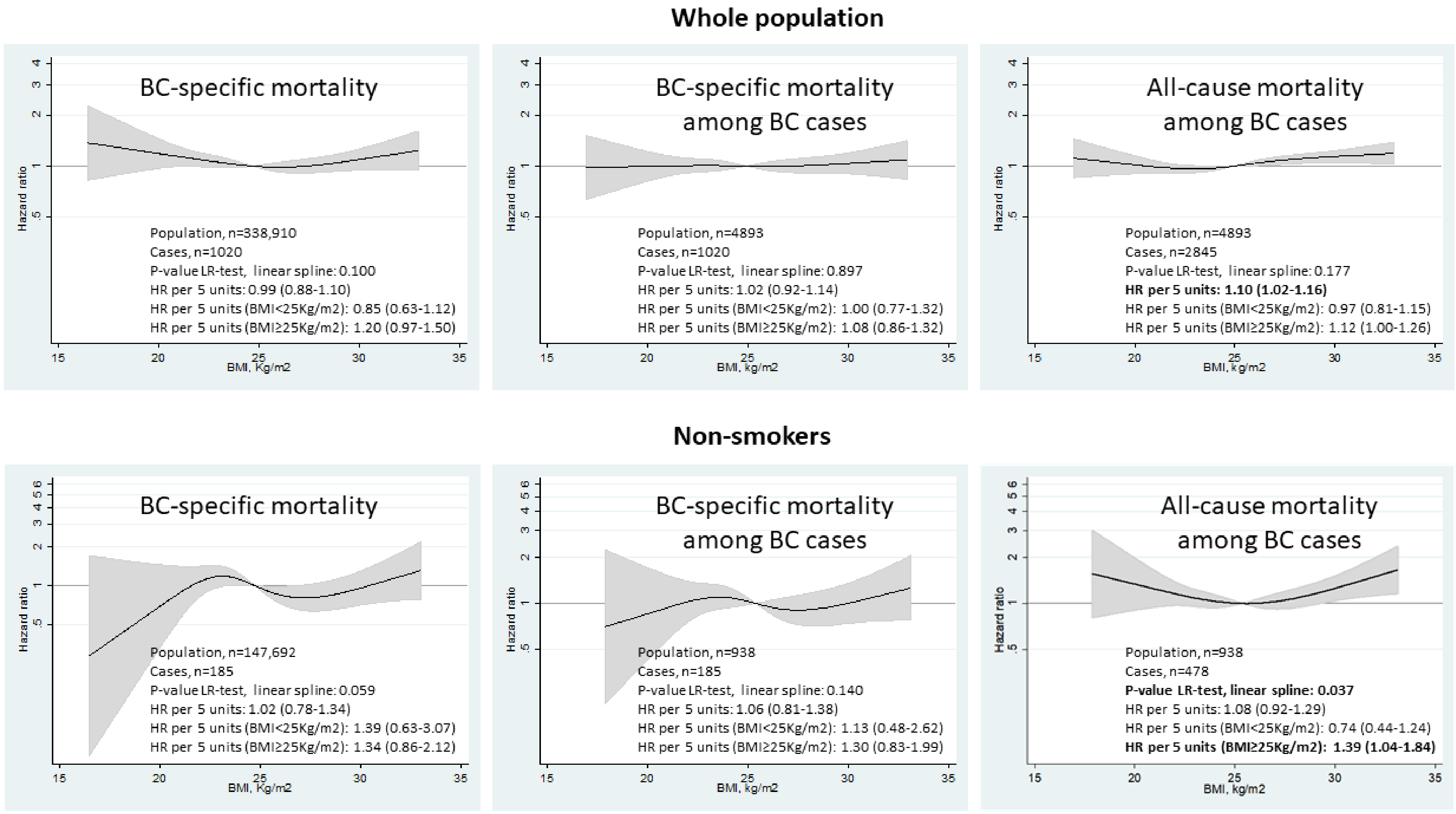

B)

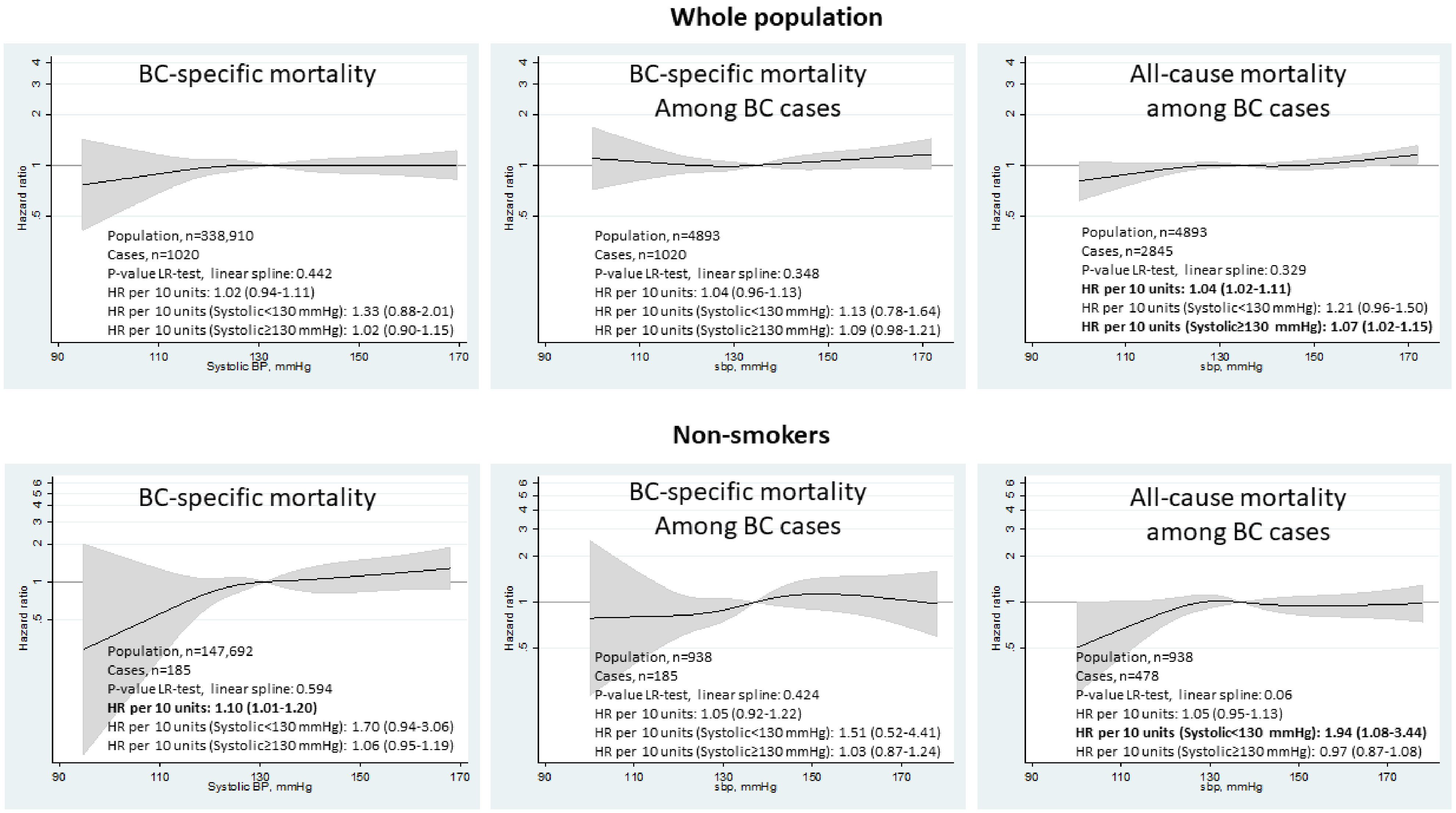
Whole population
BC-specific mortality
Population, n=338,910
Cases, n=1020
P-value LR-test, linear spline: 0.442
HR per 10 units: 1.02 (0.94-1.11)
HR per 10 units (Systolic<130 mmHg): 1.33 (0.88-2.01)
HR per 10 units (Systolic≥130 mmHg): 1.02 (0.90-1.15)
Hazard ratio
Systolic BP, mmHg
BC-specific mortality
Among BC cases
Population, n=4893
Cases, n=1020
P-value LR-test, linear spline: 0.348
HR per 10 units: 1.04 (0.96-1.13)
HR per 10 units (Systolic<130 mmHg): 1.13 (0.78-1.64)
HR per 10 units (Systolic≥130 mmHg): 1.09 (0.98-1.21)
Hazard ratio
sbp, mmHg
All-cause mortality
among BC cases
Population, n=4893
Cases, n=2845
P-value LR-test, linear spline: 0.329
HR per 10 units: 1.04 (1.02-1.11)
HR per 10 units (Systolic<130 mmHg): 1.21 (0.96-1.50)
HR per 10 units (Systolic≥130 mmHg): 1.07 (1.02-1.15)
Hazard ratio
sbp, mmHg
Non-smokers
BC-specific mortality
Population, n=147,692
Cases, n=185
P-value LR-test, linear spline: 0.594
HR per 10 units: 1.10 (1.01-1.20)
HR per 10 units (Systolic<130 mmHg): 1.70 (0.94-3.06)
HR per 10 units (Systolic≥130 mmHg): 1.06 (0.95-1.19)
Hazard ratio
Systolic BP, mmHg
BC-specific mortality
Among BC cases
Population, n=938
Cases, n=185
P-value LR-test, linear spline: 0.424
HR per 10 units: 1.05 (0.92-1.22)
HR per 10 units (Systolic<130 mmHg): 1.51 (0.52-4.41)
HR per 10 units (Systolic≥130 mmHg): 1.03 (0.87-1.24)
Hazard ratio
sbp, mmHg
All-cause mortality
among BC cases
Population, n=938
Cases, n=478
P-value LR-test, linear spline: 0.06
HR per 10 units: 1.05 (0.95-1.13)
HR per 10 units (Systolic<130 mmHg): 1.94 (1.08-3.44)
HR per 10 units (Systolic≥130 mmHg): 0.97 (0.87-1.08)
Hazard ratio
sbp, mmHg

Figure S5: The Hazard ratio (HR) (black line) and 95% confidence interval (shaded area) of BC outcomes by per 10 mmHg of diastolic blood pressure among men in the full population (A) and among never-smokers only (B). Models were derived from restricted cubic spline regression, with knots placed at percentiles of 5, 35, 65, and 95. Participants who had values that were more extreme than the equivalent of +/-2.5 standard deviations (SD) were excluded from the analyses. P value LR test, linear-spline, refers to likelihood-ratio (LR) tests of the linear model nested in a model with the addition of splines.

A)

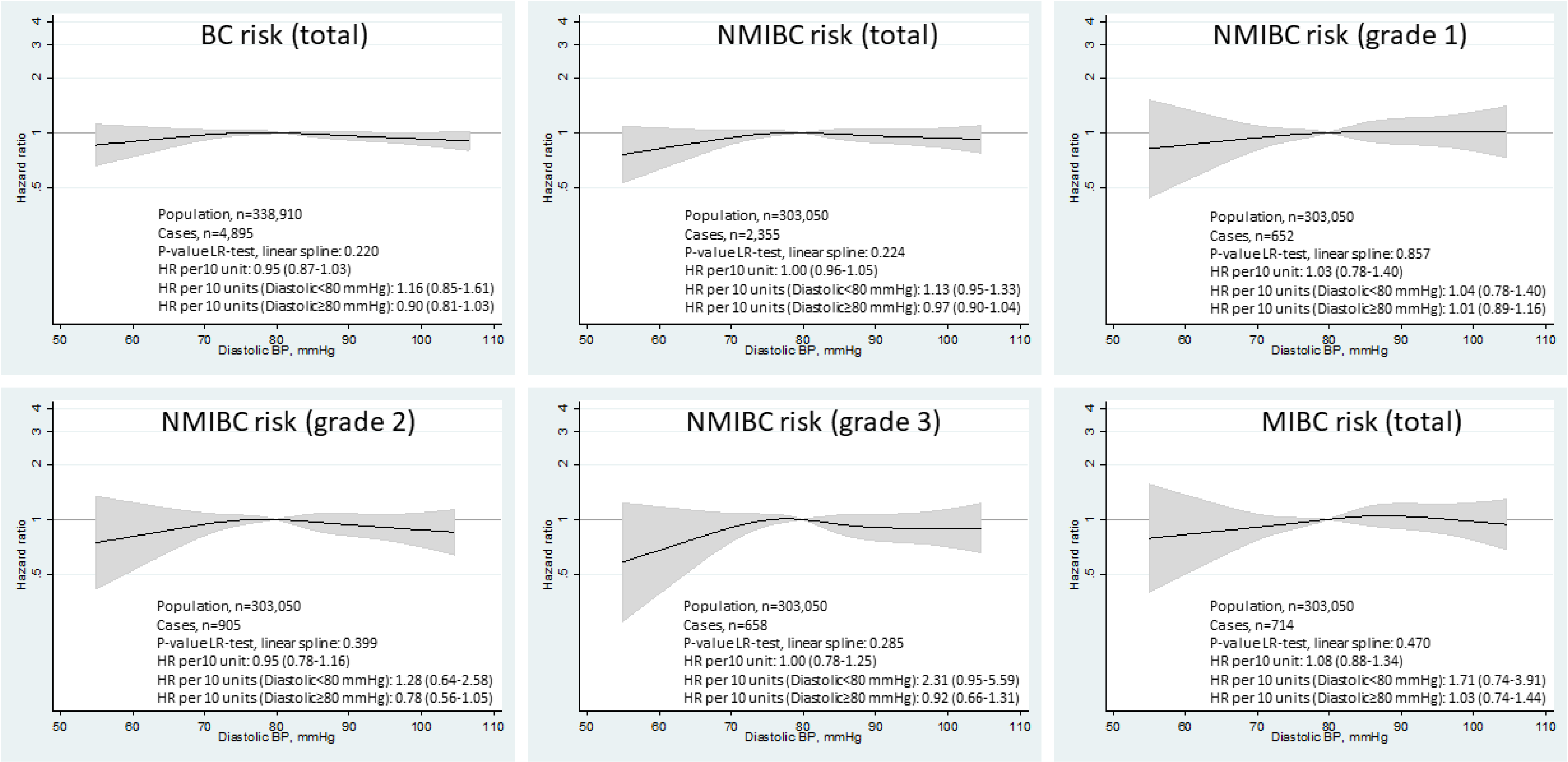

B)

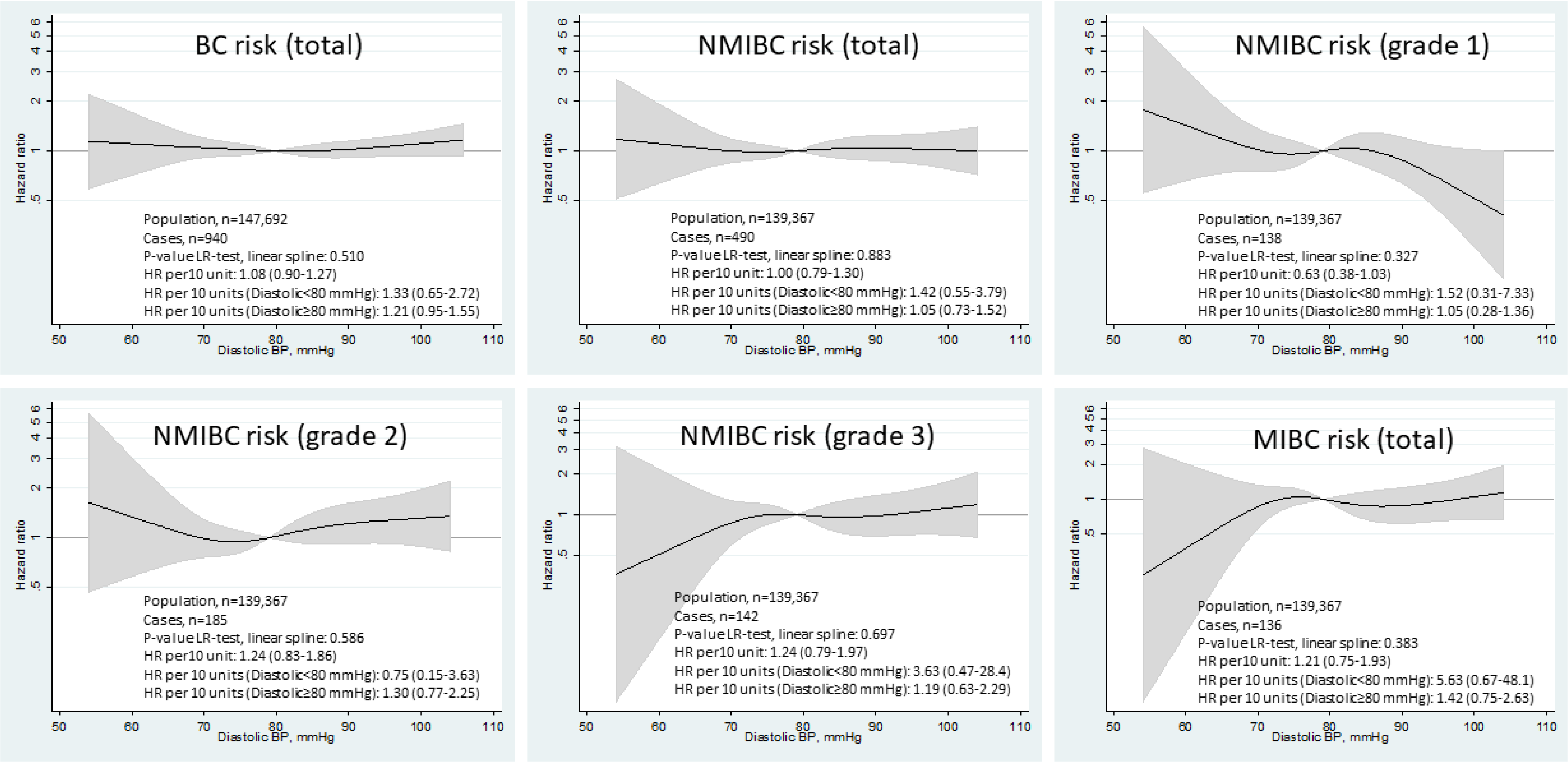
BC risk (total)
Population, n=147,692
Cases, n=940
P-value LR-test, linear spline: 0.510
HR per10 unit: 1.08 (0.90-1.27)
HR per 10 units (Diastolic<80 mmHg): 1.33 (0.65-2.72)
HR per 10 units (Diastolic≥80 mmHg): 1.21 (0.95-1.55)
NMIBC risk (total)
Population, n=139,367
Cases, n=490
P-value LR-test, linear spline: 0.883
HR per10 unit: 1.00 (0.79-1.30)
HR per 10 units (Diastolic<80 mmHg): 1.42 (0.55-3.79)
HR per 10 units (Diastolic≥80 mmHg): 1.05 (0.73-1.52)
NMIBC risk (grade 1)
Population, n=139,367
Cases, n=138
P-value LR-test, linear spline: 0.327
HR per10 unit: 0.63 (0.38-1.03)
HR per 10 units (Diastolic<80 mmHg): 1.52 (0.31-7.33)
HR per 10 units (Diastolic≥80 mmHg): 1.05 (0.28-1.36)
NMIBC risk (grade 2)
Population, n=139,367
Cases, n=185
P-value LR-test, linear spline: 0.586
HR per10 unit: 1.24 (0.83-1.86)
HR per 10 units (Diastolic<80 mmHg): 0.75 (0.15-3.63)
HR per 10 units (Diastolic≥80 mmHg): 1.30 (0.77-2.25)
NMIBC risk (grade 3)
Population, n=139,367
Cases, n=142
P-value LR-test, linear spline: 0.697
HR per10 unit: 1.24 (0.79-1.97)
HR per 10 units (Diastolic<80 mmHg): 3.63 (0.47-28.4)
HR per 10 units (Diastolic≥80 mmHg): 1.19 (0.63-2.29)
MIBC risk (total)
Population, n=139,367
Cases, n=136
P-value LR-test, linear spline: 0.383
HR per10 unit: 1.21 (0.75-1.93)
HR per 10 units (Diastolic<80 mmHg): 5.63 (0.67-48.1)
HR per 10 units (Diastolic≥80 mmHg): 1.42 (0.75-2.63)
Hazard ratio
Diastolic BP, mmHg
50 60 70 80 90 100 110
.5 1 2 3 4 5 6

Figure S6: The Hazard ratio (HR) (black line) and 95% confidence interval (shaded area) of BC-specific mortality and all-cause mortality by per10 mmHg of diastolic blood pressure among men in the full population and among never-smokers only. Models were derived from restricted cubic spline regression, with knots placed at percentiles of 5, 35, 65, and 95. Participants who had values that were more extreme than the equivalent of +/-2.5 standard deviations (SD) were excluded from the analyses. P value LR test, linear-spline, refers to likelihood-ratio (LR) tests of the linear model nested in a model with the addition of splines.

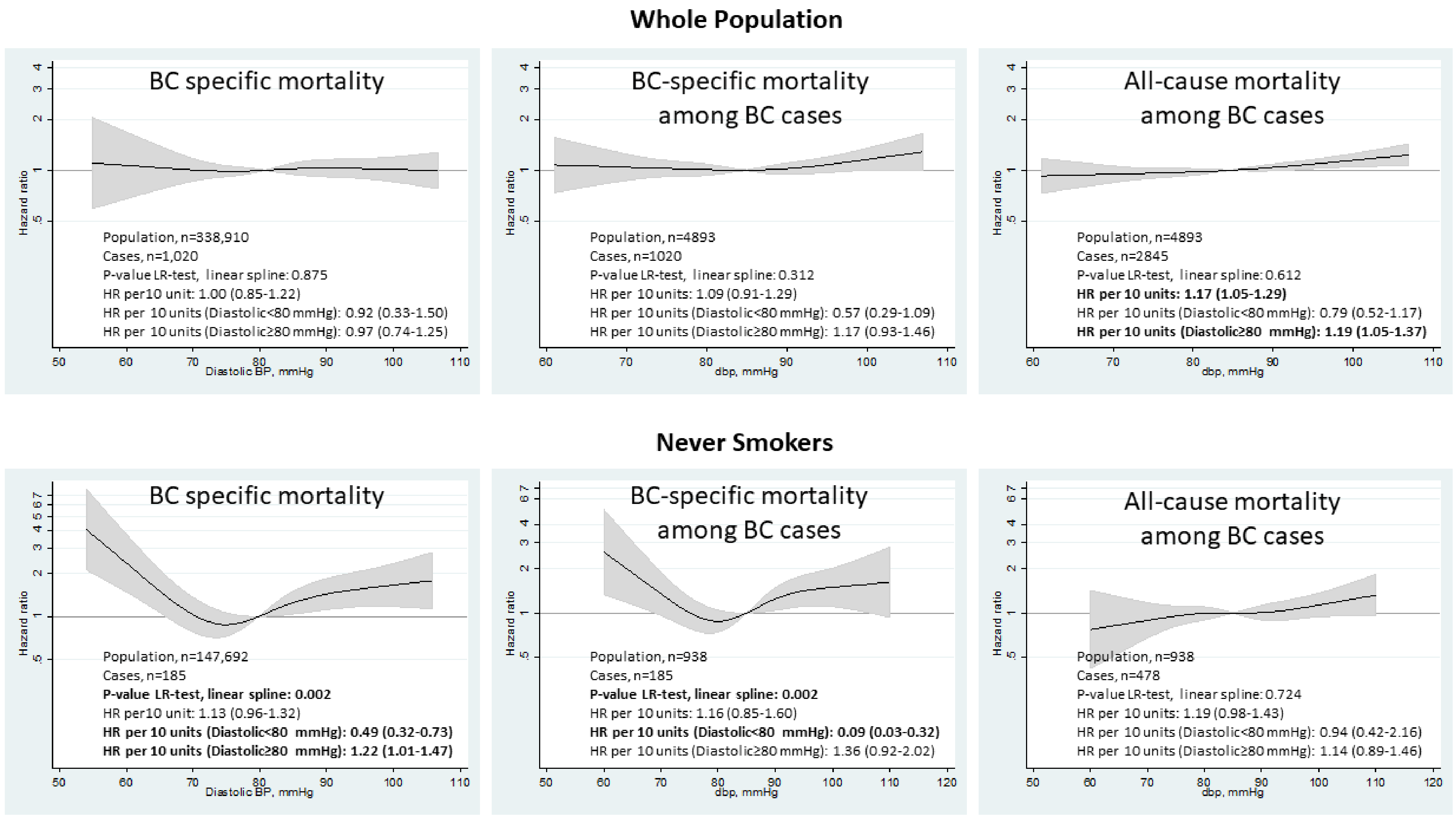

Table S1: Codebook categorical variables used in the models

Variable	Categories
Education	1. Pre-upper secondary school education <9 years.<br>2. Pre-upper secondary school 9 years.<br>3. Maximum 2 years upper secondary school.<br>4. Three years upper secondary school.<br>5. Post-upper secondary school <3 years.<br>6. Post-upper secondary school 3 years or more (excl. PhD).<br>7. PhD degree.<br>8. Missing (8%).
Date of birth	1. Before 1935<br>2. 1935-1940<br>3. 1940-1945<br>4. 1945-1950<br>5. After 1950
Cohort	1. Västerbotten Intervention Programme<br>2. Malmö Preventive Project<br>3. Construction workers cohort
Smoking	1. Never-smoker.<br>2. Ex-smokers.<br>3. Current smokers
Type of Treatment	1. No treatment.<br>2. Primarily cystectomy.<br>3. Primarily systemic.<br>4. Primarily installation.<br>5. Primarily curative radiotherapy.<br>6. Other treatments.<br>7. Multiple treatments.<br>8. Missing (37%).
Charlson co-morbidity index	1. No co-morbidities<br>2. One co-morbidity<br>3. More than one co-morbidity<br>4. Missing (36%).
Stage	1. Ta-T1<br>2. T2-T4, including metastatic tumors<br>3. Missing (37%).
Grade	1. Grade 1<br>2. Grade 2<br>3. Grade 3<br>4. Missing (40%).
BMI	Quartiles

Table S2: Calculated regression dilution ratios for BMI, SBP and DBP

	BMI	SBP	DBP
All individuals (never+ex+current smokers)	0.904	0.491	0.385
Cases only (never+ex+current smokers)	0.933	0.547	0.441
Never-Smokers (all individuals)	0.918	0.511	0.398
Never-Smokers (cases only)	0.954	0.620	0.508
Ex-smokers (all individuals)	0.891	0.502	0.383
Ex-smokers (cases only)	0.922	0.596	0.441
Current-smokers (all individuals)	0.907	0.503	0.406
Current-smokers (cases only)	0.915	0.599	0.490
Ever smokers (ex+current smokers combined) (all individuals)	0.900	0.482	0.377
Ever smokers (ex+current smokers combined) (cases only)	0.927	0.551	0.444

Abbreviations: BMI, body mass index; SBP, systolic blood pressure; DBP, diastolic blood pressure.

Table S3: Characteristics of the study participants according to cohort

**Characteristic**	**VIP**	**MPP**	**CwC**	**Total**
**Population, n (%)**	**52,055 (15.3)**	**22,276 (6.6)**	**264,579 (78.1)**	**338,910 (100)**
**Baseline age, years, mean (SD)**	**46.3 (9.2)**	**43.7 (6.6)**	**36.4 (12.7)**	**38.4 (12.5)**
**Smoking status, n (%)**				
Never-smoker	31,922 (61.3)	7,588 (34.1)	108,182 (40.9)	147,692 (43.6)
Ex-smoker	11,331 (21.8)	3,568 (16.0)	49,593 (18.7)	64,492 (19.0)
Current smoker	8,802 (16.9)	11,120 (49.9)	106,804 (40.4)	126,726 (37.4)
**BMI, kg/m^2, mean (SD)**	**26.3 (3.7)**	**24.7 (3.3)**	**24.4 (3.1)**	**24.7 (3.3)**
**Category BMI, n (%)**				
>18.5	167 (0.3)	292 (1.3)	2,572 (1.0)	3,031 (0.9)
18.5-24.9	20,464 (39.3)	12,751 (57.2)	162,846 (61.6)	196,061 (57.9)
25-29.9	24,163 (46.4)	7,865 (35.3)	85,923 (32.4)	117,951 (34.8)
≥30	7,261 (14.0)	1,368 (6.1)	13,238 (5.0)	21,867 (6.4)
**Blood pressure, mm Hg, mean (SD)**				
Systolic blood pressure	**128.0 (16.0)**	**127.1 (14.9)**	**133.0 (14.7)**	**132.2 (15.1)**
Diastolic blood pressure	**79.9 (10.3)**	**85.5 (9.7)**	**81.0 (10.4)**	**80.7 (10.4)**
**Category, systolic/diastolic, n (%)**				
<140/90 mm Hg	37,563 (72.2)	12,831 (57.6)	162,481 (61.4)	212,875 (62.8)
140/90-159/99 mm Hg	10,728 (20.6)	6,964 (31.3)	81,918 (31.0)	99,610 (29.4)
≥160/100 mm Hg	3,764 (7.2)	2,481 (11.1)	20,180 (7.6)	26,425 (7.8)
**Incident cases of BC overall, n (%)**	**353 (7.2)**	**699 (14.3)**	**3,843 (78.5)**	**4,895 (100)**
**Level of invasion, n (%)***				
Non-muscle invasive	240 (79.7)	366 (77.5)	1,673 (76.3)	2,355 (76.7)
Muscle invasive	61 (20.3)	106 (22.5)	520 (23.7)	714 (23.3)
**Grade among NMIBC, n (%) ±**				
Grade 1	76 (35.5)	88 (23.4)	518 (31.3)	682 (30.4)
Grade 2	73 (34.1)	158 (42.0)	674 (40.7)	905 (40.3)
Grade 3	65 (30.4)	130 (34.6)	463 (28.0)	658 (29.3)
**Average years of follow-up for BC risk, years, mean (SD)†**	**14.1 (7.3)**	**29.9 (8.5)**	**30.7 (8.5)**	**28.1 (10.8)**
**Average years of follow-up for BC death, years, mean (SD)§**	**14.1 (7.3)**	**30.2 (8.4)**	**30.8 (9.2)**	**28.2 (10.7)**

*Out of the 4,895 incident bladder cancer cases, staging data was available for 3,069 cases, the remaining 1,826 cases either occurred before 1997 before staging data was available or staging data was available, but stage could not be determined.

±Out of the 2,355 incident NMIBC cases grading data was available for 2,245 cases, for the remaining 110 cases, grade could not be determined.

†Follow-up until bladder cancer risk or censoring, last date of follow-up was 31 December 2014.

§Follow-up until bladder cancer death or censoring, last date of follow-up was 31 December 2014.

Abbreviations: VIP, Västerbotten Intervention project; MPP, Malmö Preventive Project; CwC, Construction Workers Cohort; SD, standard deviation; BMI, body mass index; BC, bladder cancer; NMIBC, non-muscle invasive BC.

Table S4: Hazard ratio (95% confidence interval) of bladder cancer outcomes for of systolic and diastolic blood pressure, and BMI among men in the whole population

			**BC risk Overall**		**NMIBC**		**MIBC**		**BC-specific death**
			**N=338,910 (2,895 cases)**		**N=303,050 (2,355 cases)**		**N=303,050 (714 cases)**		**N=338,910 (1,020 cases)**
**Exposure**	**Categories**	**N of cases**	**HR (95% CI)**	**N of cases**	**HR (95% CI)**	**N of cases**	**HR (95% CI)**	**N of cases**	**HR (95% CI)**
**BMI, Kg/m²**	<20 Kg/m²	190	1.00	94	1.00	29	1.00	39	1.00
	20-22.49 Kg/m²	912	0.98 (0.83-1.17)	463	1.01 (0.79-1.29)	139	0.93 (0.60-1.46)	180	0.89 (0.61-1.30)
	22.5-24.99 Kg/m²	1,572	0.96 (0.80-1.13)	732	0.93 (0.73-1.18)	245	0.92 (0.60-1.42)	338	0.89 (0.61-1.28)
	25-27.49 Kg/m²	1,309	0.95 (0.79-1.11)	623	0.99 (0.78-1.27)	176	0.79 (0.51-1.22)	273	0.81 (0.56-1.18)
	27.5-29.99 Kg/m²	576	0.96 (0.79-1.15)	277	1.07 (0.83-1.39)	83	0.89 (0.56-1.43)	124	0.84 (0.56-1.26)
	≥30 Kg/m²	336	1.11 (0.91-1.36)	166	1.30 (0.98-1.72)	42	0.91 (0.54-1.56)	66	0.95 (0.61-1.46)
	p-trend		0.396		**0.016**		0.441		0.641
**SBP, mmHg**	<120 mmHg	517	1.00	304	1.00	59	1.00	82	1.00
	120-129 mmHg	1,143	1.02 (0.83-1.26)	601	0.86 (0.65-1.15)	196	**2.47 (1.35-4.53)**	241	**1.73 (1.04-2.91)**
	130-139 mmHg	1,408	1.17 (0.96-1.45)	727	1.08 (0.83-1.45)	227	**2.77 (1.52-5.03)**	266	1.43 (0.86-2.41)
	140-149 mmHg	950	1.02 (0.81-1.28)	424	0.84 (0.62-1.15)	125	1.84 (0.96-3.54)	199	1.38 (0.81-2.38)
	150-159 mmHg	488	1.04 (0.81-1.35)	174	0.77 (0.53-1.15)	64	**2.47 (1.17-5.22)**	127	1.79 (0.98-2.95)
	≥160 mmHg	389	1.00 (0.74-1.33)	125	1.00 (0.65-1.58)	43	**2.60 (1.13-6.05)**	105	1.63 (0.86-3.09)
	p-trend (C1-C6)		0.840		0.625		0.201		0.302
**DBP, mmHg**	<70 mmHg	183	1.00	103	1.00	29	1.00	27	1.00
	70-79 mmHg	1,056	1.14 (0.76-1.71)	566	1.31 (0.76-2.27)	163	1.14 (0.41-3.17)	188	1.16 (0.41-3.34)
	80-89 mmHg	2,105	1.11 (0.74-1.68)	1 037	1.31 (0.76-2.27)	316	1.19 (0.44-3.28)	448	1.44 (0.51-3.97)
	90-99 mmHg	1,240	1.08 (0.72-1.68)	534	1.37 (0.76-2.40)	174	1.31 (0.46-3.79)	275	1.22 (0.43-3.50)
	≥ 100 mmHg	311	0.88 (0.53-1.44)	115	1.05 (0.53-2.18)	32	0.72 (0.18-2.77)	82	1.57 (0.49-5.01)
	p-trend (C1-C5)		0.328		0.880		0.910		0.568

Age was used as the underlying time metric.

Models were adjusted for date of birth, age at baseline, BMI (except for BMI), smoking status, education and cohort.

Table S5: Hazard ratio (95% confidence interval) of bladder cancer outcomes for of systolic and diastolic blood pressure, and BMI among men that were never-smokers.

			**BC risk Overall**	**N (cases)**	**NMIBC**		**MIBC**		**BC-specific death**
			**N=147,692 (940 cases)**		**N=139,367 (490 cases)**		**N=139,367 (136 cases)**		**N=147,692 (185 cases)**
**Exposure**	**Categories**	**N of cases**	**HR (95% CI)**	**N of cases**	**HR (95% CI)**	**N of cases**	**HR (95% CI)**	**N of cases**	**HR (95% CI)**
**BMI, 20 Kg/m^2**	<20 Kg/m^2	15	1.00	7	1.00	4	1.00	3	1.00
	20-22.49 Kg/m^2	141	1.30 (0.80-2.53)	86	1.94 (0.84-4.50)	14	0.47 (0.15-1.59)	20	0.90 (0.24-3.38)
	22.5-24.99 Kg/m^2	305	1.41 (0.80-2.48)	150	1.61 (0.70-3.68)	49	0.78 (0.26-2.40)	72	1.43 (0.41-5.06)
	25-27.49 Kg/m^2	276	1.36 (0.77-2.42)	140	1.74 (0.75-3.99)	39	0.68 (0.22-2.09)	52	1.03 (0.29-3.72)
	27.5-29.99 Kg/m^2	129	1.38 (0.77-2.48)	62	1.77 (0.75-4.18)	19	0.73 (0.22-2.42)	23	0.96 (0.26-3.62)
	≥30 Kg/m^2	74	1.54 (0.84-2.86)	45	**2.61 (1.09-6.25)**	11	0.86 (0.24-3.01)	15	1.39 (0.36-5.51)
	p-trend (C1-C6)		0.503		0.076		0.525		0.977
**SBP, mmHg**	<120 mmHg	90	1.00	59	1.00	9	1.00	9	1.00
	120-129 mmHg	190	0.91 (0.56-1.43)	117	0.83 (0.44-1.55)	26	1.80 (0.41-8.03)	37	2.73 (0.65-11.5)
	130-139 mmHg	279	1.41 (0.87-2.27)	150	1.06 (0.59-1.96)	47	**4.60 (1.10-19.1)**	50	3.26 (0.80-13.4)
	140-149 mmHg	143	0.94 (0.57-1.60)	92	0.81 (0.42-1.57)	25	2.57 (0.56-11.9)	32	2.18 (0.50-9.68)
	150-159 mmHg	102	1.21 (0.68-2.15)	40	0.74 (0.32-1.67)	13	2.99 (0.54-16.7)	28	**5.19 (1.12-23.8)**
	160 mmHg	106	1.62 (0.91-2.96)	32	0.91 (0.37-2.21)	16	**7.82 (1.45-42.1)**	29	**6.43 (1.36-30.1)**
	p-trend (C1-C6)		0.084		0.685		**0.025**		**0.020**
**DBP, mmHg**	<70 mmHg	28	1.00	18	1.00	1	1.00	5	1.00
	70-79 mmHg	194	1.90 (0.69-5.15)	111	1.68 (0.47-5.92)	33	97.9 (0.67-14k)	25	0.50 (0.05-5.78)
	80-89 mmHg	360	1.24 (0.45-3.31)	201	1.24 (0.37-4.32)	51	43.5 (0.29-6k)	69	0.65 (0.07-6.84)
	90-99 mmHg	279	1.82 (0.65-5.02)	132	1.65 (0.45-6.00)	41	82.5 (0.54-12k)	65	1.27 (0.12-13.7)
	≥ 100 mmHg	79	1.52 (0.49-4.69)	28	0.98 (0.20-4.57)	10	63.0 (0.34-11k)	21	1.61 (0.13-21.0)
	p-trend (C1-C5)		0.721		0.832		0.537		0.085

Age was used as the underlying time metric

Models were adjusted for date of birth, age at baseline, BMI (except for BMI), education and cohort.
